# Distinct amino acid signatures and neutralization sensitivity of plasma- and CSF-derived HIV-1 subtype C clones

**DOI:** 10.1186/s12977-026-00683-8

**Published:** 2026-08-02

**Authors:** Silondoloze Mtanjana, Nombali Gumede, Nelisiwe Zikhali, Ntokozo Ntshangase, Suhina Sirksson, Katlego Sojane, Thumbi Ndung’u, Bongiwe Ndlovu

**Affiliations:** 1 HIV Pathogenesis Programme, The Doris Duke Medical Research Institute, University of KwaZulu-Natal, Durban, South Africa; 2 Africa Health Research Institute, Durban, South Africa; 3 Ragon Institute of Mass General Brigham, Massachusetts Institute of Technology and Harvard University, Cambridge, MA USA; 4 Division of Infection and Immunity, University College London, London, UK

## Abstract

**Background:**

Broadly neutralizing antibodies (bnAbs) offer a promising alternative for HIV prevention and treatment, with the potential to cross the blood–brain barrier. However, the extent to which viral genetic variation between the peripheral blood and central nervous system impacts bnAb sensitivity remains insufficiently characterized. This study investigates sequence differences and neutralization profiles of matched plasma- and cerebrospinal fluid (CSF)-derived HIV-1 subtype C (HIV-1C) variants in patients coinfected with cryptococcal meningitis.

**Methods:**

Matched plasma and CSF samples were obtained from seven participants with HIV-cryptococcal meningitis coinfection in Durban, KwaZulu-Natal, South Africa. HIV-1C *env* was amplified, cloned into a vector, and sequenced by Sanger sequencing. Amino acid sequences within bnAb epitopes were compared between plasma- and CSF-derived clones. Additionally, we generated Env-pseudotyped viruses and assessed their neutralisation sensitivity using eight bnAbs targeting five known bnAb epitopes on HIV-1 Env.

**Results:**

Discordant bnAb escape mutations and variations in neutralization sensitivity between plasma and CSF were observed in the CD4 binding site, V2 Apex and the V3 glycan supersite. Despite these variations, VRC07-523LS demonstrated broad neutralisation activity, with no significant differences in sensitivity across plasma and CSF variants. However, only minor neutralization sensitivity variations were observed in the MPER and gp120-gp41 interface between plasma and CSF compartments.

**Conclusions:**

These findings highlight potential differences in amino acid signatures and neutralisation sensitivity between matched plasma- and CSF-derived HIV-1C variants. Our findings show that bnAb targeting the CD4-binding site (VRC07-523LS) maintain cross-compartment efficacy and have a potential in the clearance of viral reservoirs.

**Supplementary Information:**

The online version contains supplementary material available at https://doi.org/10.1186/s12977-026-00683-8.

## Introduction

The human immunodeficiency virus (HIV) remains a major global health challenge, with approximately 40.8 million people living with the virus in 2024, predominantly in Southern and Eastern Africa [1]. Despite the effectiveness of prevention strategies such as condoms, pre- and post-exposure prophylaxis, antiretroviral therapy for treatment as prevention, and voluntary medical male circumcision [2, 3]. These strategies are limited by challenges, including suboptimal adherence, restricted healthcare access, and persistent social and structural barriers [4–6]. Consequently, novel prevention and treatment approaches are urgently needed. HIV-1 establishes genetically diverse viral reservoirs across anatomical compartments, including the central nervous system, where distinct immune pressures and antiretroviral pharmacokinetics may drive compartment-specific viral evolution. Understanding how this genetic variation influences broadly neutralizing antibody sensitivity is critical for optimizing passive immunization strategies.

An HIV vaccine remains elusive due to key challenges such as the absence of immunogens capable of eliciting broadly neutralizing antibodies (bnAbs) and the extensive genetic diversity of the virus [7, 8]. Furthermore, HIV cure efforts are hindered by viral compartmentalization and the persistence of latent reservoirs [9, 10]. Despite these challenges, passive administration of bnAbs is promising in both animal models and human clinical trials as a potential treatment option [11–15]. The antibody mediated prevention (AMP) trial provided proof of concept that bnAbs can prevent HIV-1 infection, demonstrating that the VRC01 monoclonal antibody achieved 75% efficacy in neutralizing VRC01-sensitive strains [16]. Additionally, bnAbs can potentially delay viral rebound following ART interruption [14, 17, 18]. A combination of the bnAbs such as 3BNC117 and 10-1074 demonstrated extended viral suppression and reduced viral reservoirs [15, 19, 20]. These findings highlight the potential of bnAbs in both prevention and cure strategies, though further research is needed to optimize their efficacy and durability.

Previous studies established key principles of HIV-1 compartmentalization between peripheral blood and CSF, demonstrating independent evolution of viral populations under compartment-specific selective pressures [21–23]. Differences between plasma- and CSF-derived HIV-1 subtype B and C populations have been reported, with plasma viral loads generally significantly higher than those in CSF [24–26]. Notably, discordant antiretroviral drug resistance mutations between plasma and CSF-derived viruses have also been observed [26]. Analyses of HIV-1 *gag* cytotoxic T-lymphocyte (CTL) escape mutations revealed limited discordance between compartments in some participants, suggesting similar CTL selective pressures in plasma and CSF [27]. In addition, prior work from our group in individuals with cryptococcal meningitis co-infection identified differences in predicted HIV-1 subtype C coreceptor usage between compartments in a subset of participants [28]. Collectively, these studies underscore that, in some individuals, HIV-1 viral dynamics, drug resistance profiles, CTL escape patterns, and coreceptor preferences can be compartmentalized between plasma and CSF.

Furthermore, differences in neutralization sensitivity to soluble CD4 (sCD4) and bnAbs targeting the V2 apex, V3 glycan supersite, and CD4-binding site have been reported between plasma- and CSF-derived variants across multiple HIV-1 subtypes [29]. In several studies, CSF-derived viruses have shown greater neutralization resistance than plasma variants; however, such comparative analyses remain limited [29–31]. Defining these compartment-specific differences is therefore critical for informing the feasibility and optimization of bnAb-based passive immunization and HIV cure strategies.

In this study, we amplified and cloned full-length HIV-1C *env* genes from plasma and CSF samples, produced pseudoviruses, and evaluated their neutralization sensitivity against a panel of eight bnAbs targeting the CD4 binding site, V2 apex, V3 glycan, gp120-gp41 interface and membrane proximal external region (MPER) of the HIV-1 envelope trimer. We also analyzed *env* genes to identify amino acid mutations associated with bnAb resistance or sensitivity, with focus on key bnAb epitopes. Our findings provide valuable insights into genetic and neutralization differences between plasma and CSF-derived HIV-1C virus populations, which have implications for the development of effective HIV prevention and treatment strategies.

## Materials and methods

### Study participants

To determine the variations in viral populations, and neutralisation sensitivity of viruses found in peripheral blood and the central nervous system, we conducted a cross-sectional study using samples from seven participants (five males and two females). Participants had a median age of 34 years (range: 24–45 years). Participants were living with chronic HIV-1 infection, ART-naïve, and had cryptococcal meningitis based on cryptococcal antigen or India ink test using CSF (CALAS; Meridian Bioscience, Cincinnati, Ohio, USA) [32, 33]. The study that was conducted between August 2009 and March 2011 in Durban, South Africa [32, 33].

### Ethical consideration

Informed consent was obtained from patients or their guardians, and the parent study received ethical approval from the Biomedical Research and Ethics Committees of the University of KwaZulu-Natal (BF053/09), Monash University (2009001224) and the University of Western Australia (RA/4/1/2541). This sub-study also received ethical approval from the Biomedical Research and Ethics Committees of the University of KwaZulu-Natal (BREC/00005334/2023).

### Cerebrospinal fluid and plasma sample collection and processing

Paired cerebrospinal fluid (CSF) and plasma samples were collected before ART initiation. CSF samples were centrifuged at 750 ×*g* for 10 min to separate cellular debris from the fluid. The cell-free CSF and blood plasma were stored at − 80 °C within 4 h of collection [32]. Blood samples were collected in EDTA tubes and processed using Histopaque 1077 density gradient medium (Sigma-Aldrich, St. Louis, MO, USA) within 4 h of collection. Briefly, whole blood was carefully layered onto Histopaque^®^-1077 and subjected to differential centrifugation at 400 ×*g* for 30 min at room temperature, as previously described [34]. Following centrifugation, the plasma layer was carefully collected, aliquoted into sterile cryovials, and stored at − 80 °C for downstream analyses [35]. The peripheral blood mononuclear cells (PBMCs) were carefully harvested from the plasma-Ficoll interface, washed twice with phosphate-buffered saline (PBS), and cryopreserved in a freezing medium consisting of 90% fetal bovine serum (FBS) and 10% dimethyl sulfoxide (DMSO). Cryopreserved PBMCs were stored in the vapour phase of liquid nitrogen for long-term preservation [34, 35].

### HIV-1 RNA quantification

HIV-1 RNA levels were quantified in both plasma and cerebrospinal fluid (CSF) samples using quantitative real-time reverse transcriptase polymerase chain reaction (qRT-PCR) assays, following established protocols as described previously [33]. Plasma samples were collected in EDTA tubes and processed within 6 h of collection. The plasma was centrifuged and stored at − 80 °C until analysis. CSF samples were obtained via lumbar puncture and processed immediately; aliquots were stored at − 80 °C. Viral load quantification was performed with a lower limit of detection of approximately 34–50 copies/mL. The data was expressed as log_10_ copies per millilitre (log_10_ copies/mL), with undetectable samples assigned a value equivalent to half the lower detection limit for statistical analysis. Paired plasma and CSF samples were collected simultaneously from each patient to ensure accurate compartment comparison and minimize temporal variability in viral load measurements. Plasma-to-CSF HIV-1 RNA ratios were calculated to assess discordance.

### HIV-1 Env amplification from plasmid vector

Full-length HIV-1 *env* was re-amplified from pSVIII-Env plasmid vectors that were generated in a previous study using bulk PCR and cloning, as previously described [28]. For this sub-study, HIV-1 *env* was amplified using forward primer ENV A (5′-GCTTAGGCATCTCCTATGGCAGGAAGAA-3′; HXB2 positions 5945–5982), reverse primer ENV N (5′-CTGCCAATCAGGGAAGTAGCCTTGTGT-3′; HXB2 positions 9145–9171), and Phusion High-Fidelity DNA polymerase (New England Biolabs, Ipswich, MA). The amplification conditions were: 94 °C for 2 min; 45 cycles of 94 °C for 15 s, 55 °C for 30 s, 68 °C for 4 min; final extension at 68 °C for 20 min; hold at 4 °C. PCR products were analyzed by electrophoresis on 1% agarose gels stained with GelRed (Biotium, Fremont, CA) and compared against a GeneRuler 1 kb DNA ladder (Thermo Fisher Scientific). Positive amplicons were purified using the QIAquick PCR Purification kit (Qiagen, Hilden, Germany) according to the manufacturer’s instructions.

### HIV-1 *env* cloning and colony PCR

Amplified HIV-1 *env* fragments were cloned into the One-directional TOPO™ 2.1 vector (ThermoFisher Scientific Inc., Carlsbad, CA) and transformed into NEB 5-alpha competent *Escherichia coli* cells (New England Biolabs, Ipswich, MA) following the manufacturer's instructions. Transformed cells were plated on Luria–Bertani (LB) agar containing 100 µg/mL ampicillin and incubated at 37 °C overnight. Colony PCR was performed to verify the insertion of the HIV-1 *env* gene. Positive colonies were inoculated into LB broth supplemented with 100 µg/mL ampicillin and grown overnight at 37 °C with shaking at 230 rpm. According to the manufacturer’s protocol, plasmid DNA was isolated using the QIAprep Spin Miniprep Kit (Qiagen, Hilden, Germany).

### Sanger sequencing

Full-length HIV-1 *env* clones were sequenced by the Sanger method using the ABI Big Dye Terminator V3.1 cycle sequencing kit (Applied Biosystems, Foster City, CA, USA) as previously described [28, 36]. Contigs were assembled and edited using Sequencher V5.4.6 (Genecodes, Ann Arbor, MI, USA) and aligned using Clustal W. LogoSeq plots were generated using the AnalyzeAlign tool https://www.hiv.lanl.gov/content/sequence/ANALYZEALIGN/analyze_align.html.

### HIV-1 Env pseudovirus production

HIV-1 Env-pseudotyped viruses were generated in human embryonic kidney cell line (HEK 293 T/17 cells) as previously described [37–39]. Briefly, 0.5 × 10^6^ per well HEK293 T/17 cells in DMEM (supplemented with 10% FBS, HEPES, and gentamycin) were seeded in 6-well plates and incubated overnight at 37 °C, 5% CO_2_. After incubation, HEK293T/17 cells were co-transfected with recombinant Env plasmids and pSG3Δ^env^ HIV-1 backbone plasmids using X-tremeGENE™ 9 DNA Transfection Reagent (Roche Diagnostics, Mannheim, Germany) in serum-free Dulbecco’s modified eagle medium (DMEM). After 6 h, the growth medium was changed, and the cells were incubated for an additional 48 h at 37 °C and 5% CO_2_. After incubation, viral stocks were harvested, passed through a 0.45 μm filter (GVS filter technology, Stanford, USA), supplemented with 10% FBS (Gibco, Thermo Fisher Scientific, Waltham, MA, USA) and stored at − 80 °C.

### Determination of infectious dose (TCID50)

The infectivity of Env-pseudotyped viral stocks was evaluated in TZM-bl cells. Four-fold dilutions of the viral stocks were prepared in quadruplicate in Corning^®^ 96-well flat-bottom plates (Merck, Darmstadt, Germany), followed by the addition of freshly trypsinized TZM-bl cells (10,000 cells/well) containing 5 mg/ml of DEAE-dextran and the plates were incubated at 37 °C and 5% CO_2_ for 48 h (Sigma-Aldrich, St. Louis, MO, USA). After incubation, the supernatant was removed and replaced with Bright-Glo Luciferase reagent (Promega Corporation, Madison, WI, USA), and the mixture was incubated for 2 min at room temperature. After incubation, the lysate was transferred to black 96-well plates (Greiner Bio-One, Kremsmünster, Austria) and luminescence was measured using a VICTOR Nivo™ Multimode Plate Reader (PerkinElmer, Waltham, MA, USA). TZM-bl cells alone were included as a negative control, while the experimental samples were the pseudoviruses incubated with TZM-bl cells. A positive infection was defined as relative light units (RLU) 10 times greater than uninfected cell controls. The infective dose that yielded ≤ 50,000 RLUs was used for the neutralisation assays.

### TZM-bl neutralisation assay

A subset of Env amplicons was selected for cloning to evaluate neutralization sensitivity of plasma- and CSF-derived variants. Selection criteria were based on sub-clustering patterns identified through phylogenetic analysis and variations within bnAb epitopes. For participants exhibiting inter-compartmental variability, multiple sequences were selected to capture this diversity. In total, we assessed neutralization sensitivity in nine plasma-derived and seven CSF-derived clones from seven participants living with HIV-1 subtype C. The neutralization sensitivity of HIV-1 pseudoviruses derived from plasma and CSF samples was measured by Tat-regulated firefly luciferase (Luc) reporter gene expression in TZM-bl cells as previously described [39–41]. Broadly neutralizing monoclonal antibodies (bnAbs) targeting the CD4 binding site (VRC07-523LS and 3BNC117), V2-glycan (CAP256-VRC26.25, PG9 and PGM1400), V3 glycan supersite (PGT121), MPER (10E8), and the gp120-gp41 interface (PGT151) epitopes were three-fold serially diluted in growth media in 96-well flat-bottom tissue culture-treated plates. Diluted antibodies with a starting concentration of 20 μg/ml were incubated with single-round competent pseudoviruses that yields ≤ 50,000 RLUs for 1 h at 37 °C, 5% CO_2_. After incubation, TZM-bl cells (10,000/well) containing 5 mg/mL DEAE-dextran (Sigma-Aldrich, St. Louis, MO, USA) were added to the wells and incubated at 37 °C, 5% CO_2_ for 48 h. After incubation, Bright-Glo Luciferase reagent (Promega Corporation, Madison, WI, USA) was added and incubated for 2 min in the dark. The lysate was transferred to black 96-well plates (Greiner Bio-One, Kremsmünster, Austria) and luminescence was measured using a VICTOR Nivo™ Multimode Plate Reader (PerkinElmer, Waltham, MA, USA). Neutralization sensitivity was quantified as the IC50 (the antibody concentration required to achieve 50% inhibition of viral infection). Neutralization sensitivity (IC_50_ titers) was compared between blood and CSF-derived viruses. Uninfected TZM-bl cells were used as a negative control, while the virus incubated with the cells was used as a positive control. Experiments were performed at least twice, and the average IC_50_ titers were calculated.

### Statistical analysis

Data analysis was performed using GraphPad Prism version 10. To compare the frequency of amino acid residues between plasma- and CSF-derived sequences, Fisher’s exact test (two-tailed) was used to determine the differences in amino acid frequency in important positions. In addition, Fisher’s exact test was used to compare the percentage of plasma- and CSF-derived viral clones sensitive to each broadly neutralizing antibody (bnAb). For amino acid signature analyses, the full *env* sequence dataset was used (plasma n = 76; CSF n = 64 sequences from all seven participants). A non-parametric paired *t*-test was used to compare viral loads and neutralization IC_50_ titers between plasma and CSF variants. A *p* value of less than 0.05 was considered a significant difference. The association between plasma and CFS viral loads was determined using Pearson’s correlation coefficient test.

## Results

### Clinical characteristics

Seven participants (five males and two females) with a median age of 34 years (range: 24–45 years) were included in this study (Table 1). The median CD4 + T-cell count was extremely low, at 20 cells/μL (range, 5–172 cells/μL), indicating substantial immunosuppression in most individuals. The median plasma HIV-1 viral load was log_10_ 4.94 copies/mL (range: 4.58–5.67 log_10_ copies/mL), while the median CSF viral load was log_10_ 4.83 copies/mL (range: 2.52–5.85 log_10_ copies/mL). There was no statistically significant difference between median plasma and CSF HIV-1 viral loads, and only a weak, non-significant positive correlation was observed in viral loads between plasma and CSF samples (Pearson’s r = 0.32, *p* = 0.49) (S1 Fig). Pleocytosis (defined as greater than five white blood cells (WBCs) in the CSF) was detected in six of the seven participants, while the CSF WBC counts ranged from 28 to 154 cells/μl. One participant (CM032) showed no pleocytosis, although the absence of WBCs does not rule out underlying neuroinflammation or blood–brain barrier disruption.

**Table 1:** Demographic and clinical characteristics of the study participants

Patient ID	Age	Sex	CD4 count (cells/μl)	Plasma VL (Log_10_ copies/ml)	CSF VL^a^ (Log_10_ copies/ml)	CSF WBC (cells/μl)
CM019	45	M	7	5.15	4.33	28
CM032	34	F	5	4.94	2.52	0
CM052	24	F	172	4.62	4.83	36
CM089	27	M	114	5.67	5.85	154
CM098	40	M	14	4.76	5.27	66
CM108	27	M	20	5.02	5.02	28
CM132	34	M	53	4.58	4.43	38
Median	34		20	4.94	4.83	36

^a^Plasma VL and CSF VL were compared using the non-parametric paired *t-*test

### Differences in HIV-1 *env* amino acid signatures between plasma- and CSF-derived viruses

**Fig. 1:**
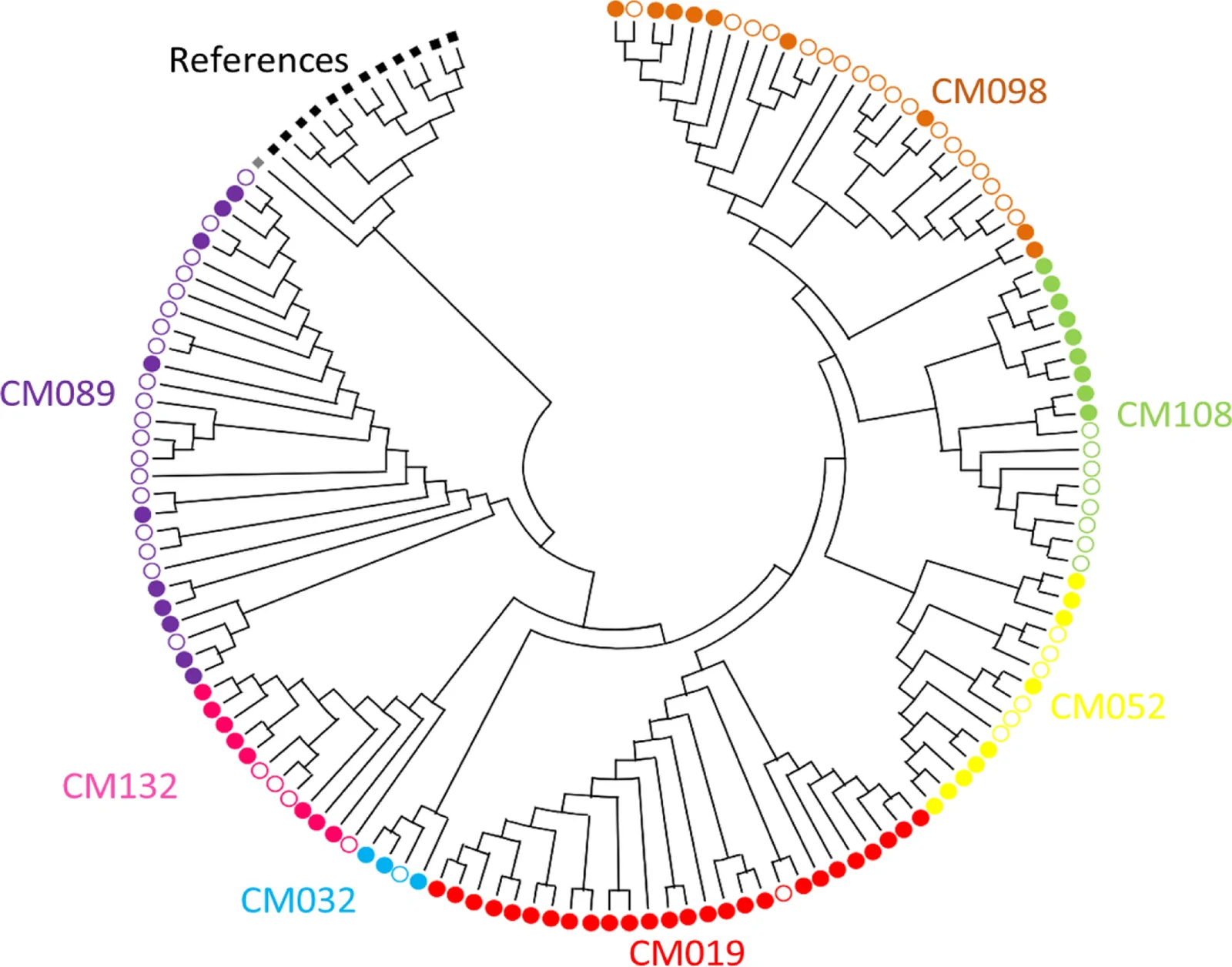
Maximum-likelihood phylogenetic relatedness of HIV-1 env sequences obtained from plasma and cerebrospinal fluid (CSF) samples. The phylogenetic tree was constructed using the GENEIOUS software and was rooted to ref C (consensus sequence) along with the 75 plasma and 64 CSF patients derived *env.* Mega software was used to assign individual colours for each participant and annotate and finalize the tree. HIV-1 *env* sequences produced from plasma are shown in closed circles, while sequences produced from CSF samples are shown in open circles, and the ref A, B, D, F2, G, CRF01_AE, CRF02_AG, CRF15_1B, CRF33_1B, CRF34_1B are shown in closed black squares and ref C is shown in a closed grey square

We sought to identify differences in HIV-1 *env* amino acid signatures between 76 plasma- and 64 cerebrospinal fluid (CSF)-derived sequences, with a particular focus on residues implicated in sensitivity to bnAbs (Fig. 1). Our primary analysis focused on key sites within the CD4 binding site (CD4bs), specifically the N-linked glycosylation sites at positions 197, 234, 276, and amino acids at positions 279, 368, and 456 (Fig. 1A). Amino acid motif corresponding to a PNGS at position 197 was highly conserved (100%) in both plasma and CSF-derived sequences.

In contrast, the frequency of the amino acid motif corresponding to a PNGS in position 234 was significantly higher in plasma compared to CSF sequences (*p* = 0.006). Approximately 48/76 (63.2%) of plasma-derived sequences maintained the intact PNGS at this position, while 36.8% showed mutations including asparagine (N), aspartic acid (D), or serine (S). In the CSF compartment, only 25/64 (39.1%) of sequences retained the N234 PNGS, whereas 60.9% exhibited mutations, including aspartic acid (D), asparagine (N), or lysine (K) (Supp Table S1).

Significant amino acid variations were also observed at position N276 (*p* = 0.003) (Supp Table S1); 62/76 (81.6%) of plasma-derived sequences had a conserved PNGS, while 18.4% displayed mutations involving asparagine (N) or glutamic acid (E). Only 37/64 (57.8%) of CSF-derived sequences retained the PNGS, and 42.2% had mutations, including aspartic acid (D) or asparagine (N) residues (Fig. 1A). We further examined position 279, and noted variations in amino acid signatures (*p* = 0.037): 52/76 (68.4%) of plasma-derived sequences had aspartic acid (D), and 31.6% had asparagine (N). In contrast, 54/64 (84.4%) of CSF-derived sequences presented with aspartic acid (D), while only 15.6% harboured asparagine (N) mutations (Fig. 1A) (Supp Table S1). Additionally, we analysed amino acid residues at position 368, both plasma- and CSF-derived sequences consistently retained aspartic acid (D) at this position, aligning with previous reports highlighting its importance for maintaining the structural integrity and electrostatic interactions essential for CD4 binding [42, 43].

Finally, analysis of position 456 revealed no significant differences in amino acid signatures (*p* = 0.161); 62/76 (81.6%) of plasma-derived sequences contained arginine (R), with 18.4% presenting mutations including a tryptophan (W), histidine (H), or tyrosine (Y). In CSF-derived sequences, 45/64 (70.3%) retained arginine (R), while 29.7% exhibited tryptophan (W) or histidine (H) mutations (Fig. 1A). These findings demonstrate significant variations in the frequency of amino acid mutations at positions 234, 276, and 279 within the CD4 binding site between plasma and CSF-derived sequences.

### Higher frequency of escape mutations in plasma-derived *env* sequences compared to CSF-derived sequences in the V2 apex

We analyzed the N-linked glycosylation sites in the V2 apex at positions 156 and 160, as well as amino acids at position 164, 166, and 169 (Fig. 1B). Significant variations in the amino acid motif corresponding to a PNGS residues were observed at position 156 (*p* = 0.004) (Supp Table S1); 47/76 (61.8%) of plasma-derived sequences retained the intact PNGS, while 38.2% had either a glutamine (Q), lysine (K), or serine (S) mutations. In contrast, 54/64 (84.4%) of CSF-derived sequences retained the PNGS, while 15.6% had a serine (S) or glutamine (Q) mutation. The N160 PNGS was conserved in 60/76 (78.9%) of plasma sequences, while 21.1% had a lysine (K) or tyrosine (Y) mutation, whereas all 64/64 (100%) of CSF-derived sequences had a conserved N160 PNGS (*p* < 0.0001). Additionally, the frequency of glutamic acid (E) was significantly higher in CSF compared to plasma at position 164 (*p* < 0.0001); 49/76 (64.5%) of plasma-derived sequences had glutamic acid (E), while 35.5% had either a glycine (G), valine (V), or glutamine (Q) mutation. In CSF, 63/64 (98.4%) of sequences retained glutamic acid (E), while only 1.6% of sequences had a glycine (G) mutation.

Furthermore, arginine (R) at position 166 was highly conserved (> 99%) in both Plasma and CSF variants, with only 1% of plasma sequences showing a glutamine (Q) mutation. Lastly, the frequency of lysine or arginine at position 169 was higher in CSF than in plasma sequences (*p* = 0.037). Approximately 65/76 (85.5%) of plasma sequences had the R/K residue, with 14.5% harbouring escape mutations including glutamic acid (E) and asparagine (N), while the majority [62/64 (96.9%)] of CSF sequences had the R/K residue, and only one (3.1%) sequence harboured asparagine (N) mutation. Plasma-derived sequences had a higher frequency of escape mutations in the V2 loop than CSF-derived sequences; these findings suggest that V2-apex bnAb resistance might be slightly higher in plasma than in the CSF compartment.

### Variations in the frequency of mutations in the V3 glycan supersite between plasma- and CSF-derived sequences

We compared amino acid signatures within the V3 glycan supersite between plasma and CSF-derived sequences and observed significant differences in PNGS conservation and amino acid frequency (Fishers exact, *p* < 0.05) (Fig. 1C). Conservation of the amino acid motif corresponding to a PNGS at position 295 did not differ significantly between plasma and CSF (*p* > 0.05). The frequency of the amino acid motif corresponding to a PNGS at position 301 was significantly higher in CSF than plasma (*p* < 0.0001); 55/76 (72.4%) of plasma-derived sequences had a conserved N301 PNGS, while 27.6% had an asparagine (N) or lysine (K) mutation. In contrast, the N301 PNGS was highly conserved in the majority [63/64 (98.4%)] of CSF-derived sequences, with only one sequence (1.6%) possessing an asparagine (N) residue (Supp Table S1). Furthermore, the N332 PNGS was highly conserved in both plasma and CSF sequences; 62/76 (81.6%) of plasma sequences had the N332 glycan, while 18.4% had mutations such as an asparagine (N), threonine (T), or lysine (K) residue. In comparison, 45/64 (70.3%) of CSF-derived sequences had a conserved N332 PNGS, and 29.7% had asparagine (N), threonine (T), and isoleucine (I) substitutions. The frequency of V3-glycan supersite escape mutations was slightly higher in the CSF compared to plasma-derived sequences.

### High frequency of escape mutations in the proximal MPER, compared to the distal MPER and gp120-gp41 interface across plasma-and CSF-derived sequences

Our analysis revealed few escape mutations within the MPER and gp120-gp41 interface epitopes across both plasma- and CSF-derived sequences (Fig. 1D, E). While the proximal MPER region had numerous escape mutations, glutamic acid (E) at position 662 was absent in all sequences; both plasma and CSF sequences had alanine residues (A662), which is associated with resistance to 2F5 mAb. While aspartic acid (D664) was maintained in over 96% of sequences across both Plasma and CSF variants, and this residue is associated with high sensitivity to 2F5 mAb (Fig. 1D) [44, 45]. Furthermore, substantial variability was also observed at position 667; none of the sequences harboured the canonical alanine residues. Instead, various mutations, including asparagine (N), lysine (K), glutamine (Q), and glutamic acid (E) were observed in both plasma and CSF sequences, consistent with prior reports that HIV-1 subtype C viruses are resistant to 2F5 mAb, and they lack A667 residues [46, 47].

Within the distal MPER (positions 671–683), residues critical for 10E8 antibody sensitivity demonstrated exceptional conservation. Tryptophan at position 672 (W672) and phenylalanine at position 673 (F673) were fully conserved across all sequences, indicating intact potential for 10E8-mediated neutralization (Fig. 1D) [48, 49]. These aromatic residues are essential for 10E8 recognition, with mutations at these positions resulting in > 1000-fold reduction in binding affinity [44, 50]. At position 674, variations involved the amino acid motif corresponding to a PNGS and amino acids previously associated with reduced 10E8 sensitivity, though most sequences maintained the conserved glycan and aspartic acid residues. Notably, there were no differences in the frequency of threonine residues at position 676 (T676) in both plasma and CSF sequences, which is linked to enhanced 10E8 neutralization [44]. Positions 680 and 683 were highly conserved in both plasma and CSF sequences, with lysine (K683) being predominant, further confirming the limited escape potential within this region [48, 49].

At the gp120-gp41 interface, critical amino acid motifs corresponding to a PNGS at positions 611 (N611) and 637 (N637) were conserved across both plasma and CSF variants (Fig. 1E). These glycans are essential for broadly neutralizing antibody recognition at the gp120-gp41 interface [51]. Their conservation across both plasma and CSF variants suggests minimal selective pressure and preservation of neutralization-sensitive sites in both viral populations. These findings indicate that broadly neutralizing antibodies targeting these conserved epitopes may retain efficacy against viruses from both anatomical Plasma and CSF variants.

### Differences in neutralization sensitivities between plasma and CSF-derived clones

**Fig. 2:**
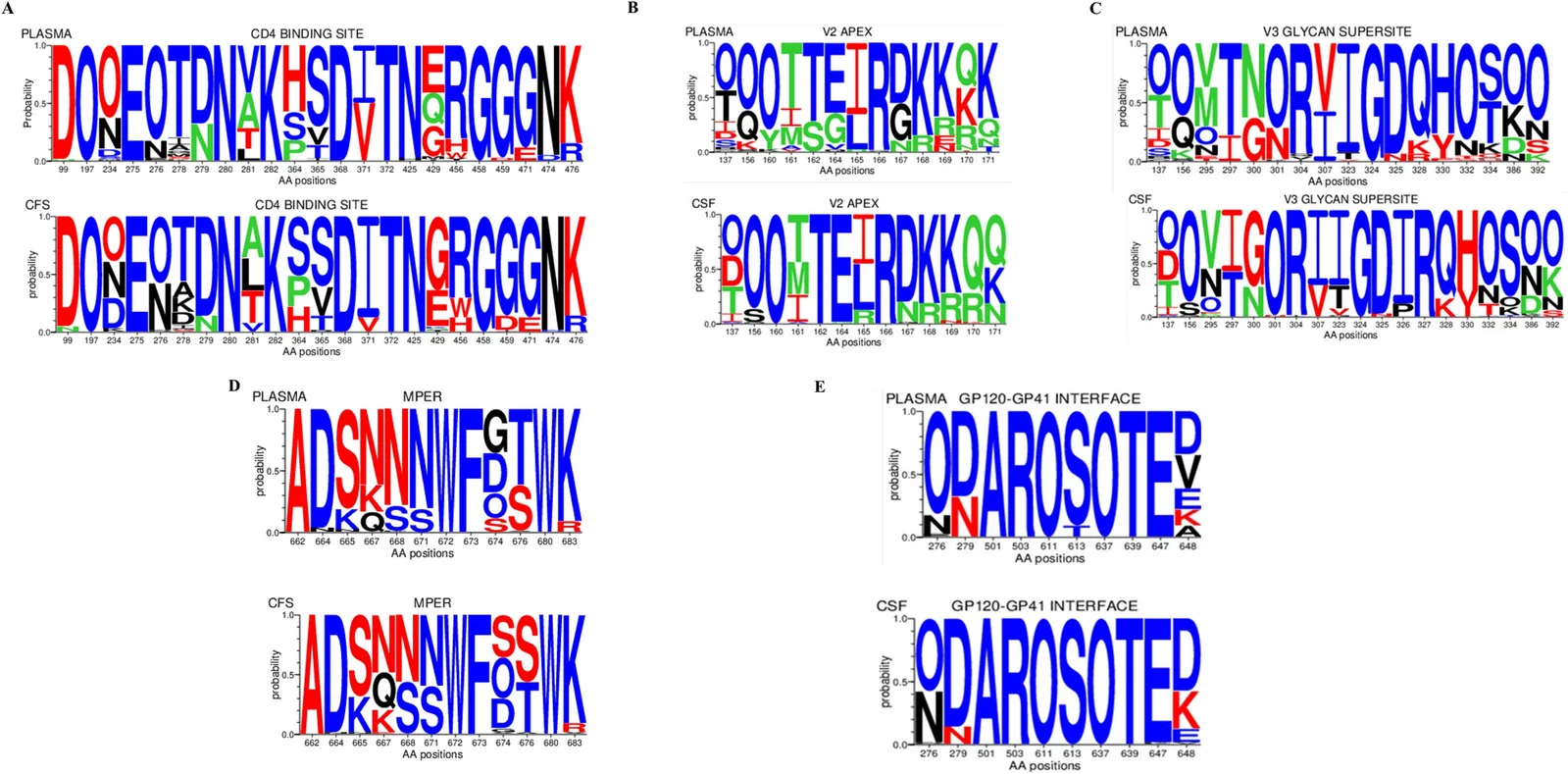
Logogram of HIV-1 clonal sequences from plasma and CSF-derived HIV-1 env sequences focusing on the CD4 Binding site (**A**), V2 apex (**B**), V3 glycan supersite (**C**), gp120-gp41 interface (**D**) and MPER (**E**). Amino acid frequency (y-axis) is shown for each residue at specific positions. The size of each AA in the logogram is proportional to its frequency, and the “O” represents N-linked glycans. Amino acid residues associated with neutralization sensitivity are shown in blue, while AA residues associated with resistance are shown in red. AA residues associated with either resistance or sensitivity to different antibodies within each class are shown in green, and residues in black are AA residues that have not been characterized

**Fig. 3:**
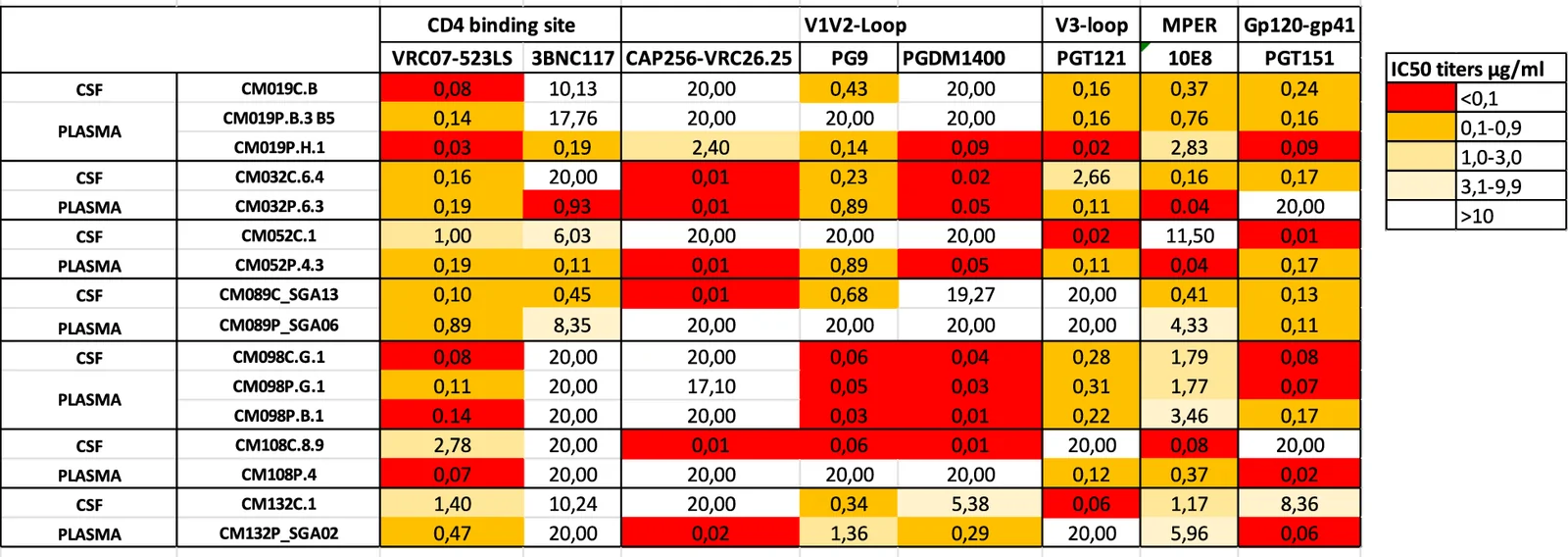
Neutralization heatmap depicts the neutralization sensitivity (IC_50_ values in µg/ml) of HIV-1 subtype C *Env*-pseudotyped viruses derived from plasma and cerebrospinal fluid (CSF). Neutralization sensitivity was measured against a panel of eight broadly neutralizing antibodies (bnAbs) targeting different epitopes: CD4 binding site (CD4bs), V2-apex, V3-glycan supersite, MPER, and gp120–gp41 interface. The colour coding reflects the neutralization potency: red indicates high sensitivity (IC_50_ < 0, 1 μg/ml), yellow indicates moderate sensitivity (IC_50_ = 1, 0–3, 0 μg/ml), cream white indicates IC_50_ > 3–9.9 and clear squares indicate no neutralization (IC_50_ > 10 μg/ml)

**Fig. 4:**
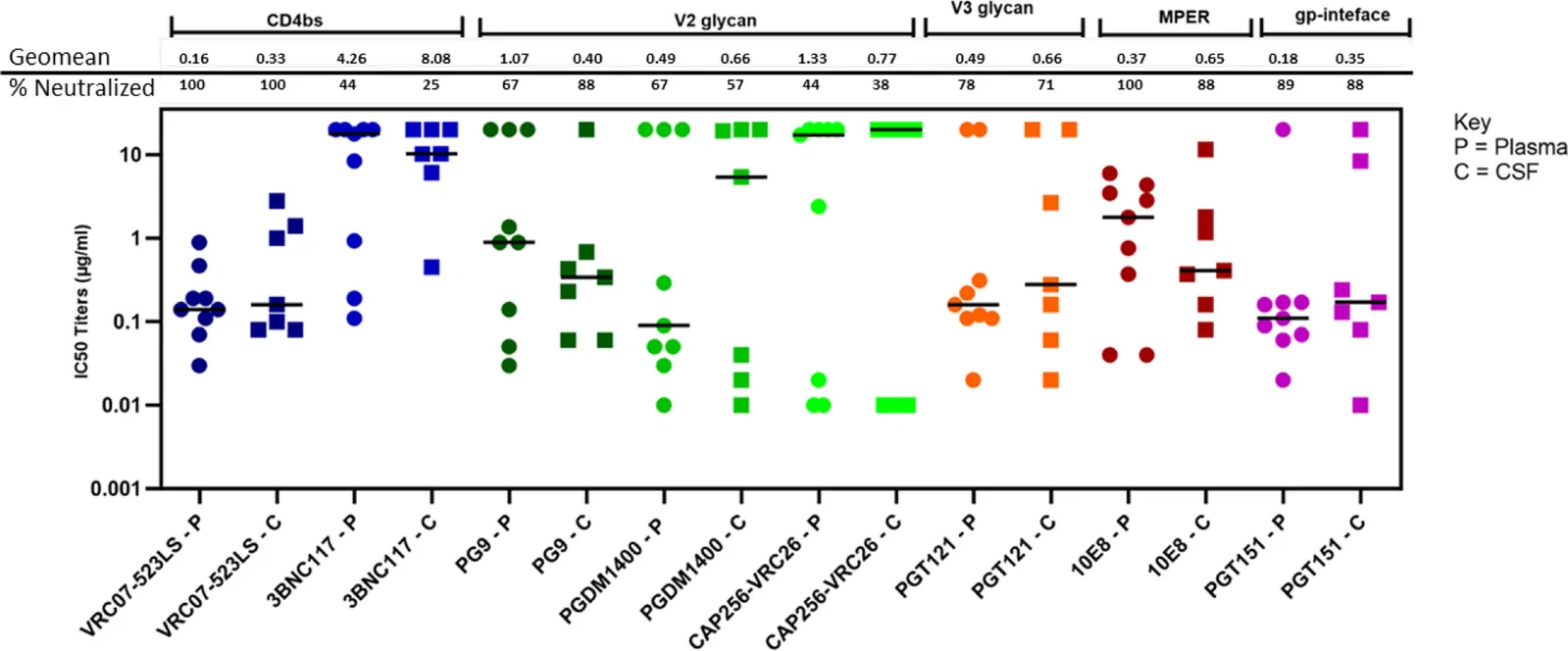
Scatter plots presentation of the neutralization sensitivity shown in IC_50_ titers. The neutralization sensitivity of HIV-1 subtype C Env-pseudotyped viruses derived from plasma and cerebrospinal fluid (CSF) to eight bnAbs targeting different epitopes: the CD4 binding site (CD4bs) (VRC07-523LS, 3BNC117), V2-apex (PG9, PGDM1400, CAP256-VRC26), V3-glycan supersite (PGT121), MPER (10E8), and the gp120-gp41 interface (PGT151). The scatter plot shows the IC50 titers (µg/ml) of each bnAb against the respective virus clones, with plasma-derived viruses represented by circles and CSF-derived viruses by squares. Black lines indicate the median IC_50_ titers

A subset of Env amplicons was selected for cloning, followed by the evaluation of neutralisation sensitivity between plasma and CSF variants. The selection of Env amplicons was based on sub-clustering observed on the phylogenetic analysis and variations within bnAb epitopes. We assessed nine plasma-derived and seven CSF-derived clones from seven participants living with HIV-1 subtype C. These clones were tested against eight bnAbs targeting distinct Env epitopes: the CD4 binding site (VRC07-523LS and 3BNC117), the V2-apex (PGDM1400, PG9, CAP256.VRC26.25), the V3 glycan supersite (PGT121), the membrane-proximal external region (MPER; 10E8), and the gp120-gp41 interface (PGT151) (Figs. 2, 3, 4).

### CD4 binding site bnAb sensitivity

Overall, VRC07-523LS demonstrated the highest neutralization coverage across all plasma- and CSF-derived clones, and there were no differences in neutralisation sensitivity (Figs. 1, 2, 3). In contrast, 3BNC117 showed high resistance in both plasma and CSF-derived clones (*p* = 0.633). Furthermore, 44% of plasma-derived clones were sensitive to 3BNC117, compared to 25% of CSF-derived clones, indicating a slightly reduced susceptibility among viruses in the CSF (Supp Table S2). This high 3BNC117 resistance could be due to the presence of escape mutations at position 456 involving histidine (H) and tryptophan (W) (Fig. 1). These mutations were previously associated with resistance to the 3BNC117 monoclonal antibody.

Detailed participant-level analysis revealed additional differences (Fig. 1). In participants CM032 and CM019, plasma-derived clones (CM032P.6.3 and CM019P.H.1) were sensitive to both VRC07-523LS and 3BNC117, while their corresponding CSF-derived clones (CM032C.6.4 and CM019C.B) retained sensitivity to VRC07-523LS but were resistant to 3BNC117. However, the second CM019 plasma clone (CM019P.B.3.B5) was only sensitive to VRC07-523LS and resistant to 3BNC117. In participant CM052, viruses derived from both Plasma and CSF variants were neutralised, although the plasma-derived clone (CM052P.4.3) had a slightly higher sensitivity to both bnAbs. In contrast, the CSF-derived clone (CM052C.1) exhibited reduced sensitivity to both bnAbs. Interestingly, the opposite was observed in participant CM089, where the CSF-derived clone (CM089C_SGA13) was sensitive to 3BNC117, while its plasma-derived counterpart (CM089P_SGA06) was resistant. Minor differences in neutralization sensitivity/potency were observed between Plasma and CSF variants for participants CM098, CM108, and CM132. These findings demonstrate that compartment-specific differences in sensitivity to 3BNC117 and VRC07-523LS are participant-dependent, but reduced susceptibility among CSF-derived viruses was frequently observed.

### V2-apex bnAb sensitivity

The V2-apex bnAbs (CAP256. VRC26.25, PG9 and PGDM1400) showed minor variations in neutralisation sensitivity patterns (*p* > 0.05) between plasma and CSF clones (Figs. 2, 3). PG9 demonstrated the highest coverage in CSF (88%) compared to plasma viruses (67%); however, the differences were not significant (*p* = 0.585). PGDM1400 neutralised 67% of plasma viruses and 57% of CSF clones. While CAP256.VRC26.25 showed lower neutralisation coverage neutralising 44% of plasma viruses, and only 38% of CSF clones (*p* > 0.05).

Participants CM052 and CM089 demonstrated a clear variation where plasma-derived clones showed V2-apex sensitivity while CSF-derived clones were predominantly resistant. CM052 showed complete CSF resistance across all three V2-apex bnAbs, while CM089 showed partial CSF resistance (sensitive only to PG9 and CAP256.VRC26.25, resistant to PGDM1400). CM108 displayed the opposite pattern, with complete plasma-derived clone resistance to all V2-apex bnAbs, while the CSF-derived clone remained sensitive to CAP256.VRC26.25 and PG9. Participants CM032, CM098, and CM132 all showed discordant neutralisation patterns, but both plasma and CSF clones retained partial sensitivity to different V2-apex bnAbs. These findings demonstrated discordant patterns of neutralization sensitivity between plasma and CSF clones.

### V3 glycan supersite bnAb sensitivity

PGT121 demonstrated high neutralization coverage in both plasma and CSF clones, neutralizing 78% of plasma-derived clones and 71% of CSF-derived clones; (*p* = 1.000) (Supp Table S2). Although participant-specific differences in sensitivity to the V3 glycan supersite bnAb PGT121 were also evident. In CM108, the plasma-derived clone (CM108P.4) was sensitive to PGT121, while the CSF-derived clone (CM108C.8.9) was resistant. An inverse pattern was observed in CM132, with the plasma-derived clone (CM132P_SGA02) resistant and the CSF-derived clone (CM132C.1) sensitive. In contrast, both plasma- and CSF-derived clones from CM089 were resistant to PGT121.

Differences in PGT121 coverage were identified in CM019, CM032, and CM052. For instance, CM019, the plasma-derived clone CM019P.H.1, was sensitive to PGT121, whereas a second plasma-derived clone from this participant (CM019P.H.2) had an IC_50_ comparable to that of CSF-derived clone CM019C.B, which showed reduced sensitivity. This within-participant heterogeneity among plasma clones highlights the difficulty in attributing differences solely to compartment of origin. CM032 also showed variability in sensitivity across Plasma and CSF variants. In contrast, no significant differences in PGT121 sensitivity were observed between plasma- and CSF-derived clones in participants CM089 and CM098. Despite this participant-level heterogeneity, PGT121 sensitivity did not significantly differ between plasma and CSF (*p* > 0.05).

### MPER and gp120-gp41 interface bnAb sensitivity

The broadly neutralizing antibody (bnAb) 10E8, targeting the MPER region, exhibited high neutralisation coverage, neutralizing all plasma-derived clones and 88% of CSF-derived clones; this difference was not statistically significant (*p* = 0.438) (Supp Table S2). While overall sensitivity was high, patterns of compartment-specific sensitivity varied markedly between individuals. Notably, participant CM052 showed a striking difference: the plasma-derived clone was highly sensitive, whereas the CSF-derived clone was resistant to 10E8. Conversely, CM108 and CM132 showed higher neutralisation sensitivity of the CSF clone compared to plasma clone. Participants CM019 and CM098 demonstrated relatively modest differences in MPER sensitivity across Plasma and CSF variants.

The gp120-gp41 interface bnAb PGT151 achieved approximately equal coverage in both plasma- and CSF-derived clones, yet also revealed participant-specific compartmentalization patterns (*p* > 0.05). CM032 showed divergent responses, plasma-derived clone was resistant, while the CSF-derived clone was highly sensitive to PGT151. CM108 displayed the reverse pattern; high sensitivity was observed in plasma clone, while resistance to PGT151 was observed in the CSF clone. Both CM052 and CM132 demonstrated quantitative rather than qualitative differences: CM052 plasma-derived clone showed reduced sensitivity compared to its CSF counterparts, while CM132 plasma-derived clone exhibited enhanced sensitivity relative to the moderately sensitive CSF-derived clones. In contrast, CM019P.H.1 and CM098P.G displayed comparable neutralisation sensitivity across both Plasma and CSF variants.

## Discussion

Phylogenetic analysis of HIV-1 *env* sequences derived from plasma and CSF Plasma and CSF variants previously revealed diverse patterns of viral diversity and compartmentalization in this cohort of participants with cryptococcal meningitis coinfection [28]. Here, we identified variations in amino acid signatures across key broadly neutralizing antibody (bnAb) epitopes, including the CD4 binding site (CD4bs), V2 apex, V3 glycan supersite, gp120-gp41 interface, and the membrane-proximal external region (MPER). These sequence differences were accompanied by notable variations in neutralization sensitivity to various classes of bnAbs.

Our findings suggest that the CD4 binding site (CD4bs) remains an accessible and effective target for neutralization, even in the context of HIV-1 evolution and compartmentalization, as evidenced by the consistent cross-neutralization of both plasma- and CSF-derived viruses by VRC07-523LS. However, we observed a high resistance to 3BNC117, particularly in CSF-derived clones. Our findings are consistent with previous studies that reported high sensitivity of southern African clade C viruses to VRC07-523LS and high resistance to 3BNC117 [52–55]. The increased resistance to 3BNC117 in our study may be driven by specific escape mutations within the CD4 binding site, including aspartic acid, serine, or lysine at position 234 (N234D/S/K), aspartic acid at position 276 (N276D), and histidine or tryptophan at position 456 (R456W/H). These single-point mutations could affect glycan shielding and induce conformational changes that restrict bnAb accessibility at the CD4 binding site. The N276D mutation was previously reported to interfere with the possible N-linked glycosylation site needed for 3BNC117 recognition [56, 57]. This aligns with emerging evidence from our group, where we reported similar resistance patterns in the FRESH and Baby Cure cohorts [55].

Interestingly, there were minor compartmental differences in the sensitivity to PGT121 mAb. This could be attributed to the glycan promiscuity of PGT121, which is not strictly dependent on the N332 glycan but can recognize a variety of amino acid motifs corresponding to a PNGS as within the V1V2 and V3 loops [59]. This flexibility may confer resilience to the glycan shifts and sequence variations observed between compartments, particularly in contrast to the pronounced compartmentalisation seen with V2-apex bnAbs, which showed significant differences in neutralisation sensitivity between plasma and CSF clones.

The discordance in neutralization sensitivity between plasma and CSF observed in our study is consistent with previous studies that characterised viral divergence between these compartments [29, 58]. Previous studies reported significant differences in the sensitivity of broadly neutralizing antibodies (bnAbs) between CSF- and blood plasma-derived HIV-1 variants across multiple patients. CSF variants showed increased resistance to PGT121, PG16, and PGT145 compared to plasma variants [29]. Specifically, the researchers sequenced full-length *env* variants from paired CSF and blood samples in individuals infected with various HIV-1 clades, including subtype C, among others. They observed significant compartmentalization and differences in neutralization sensitivity in samples from subtype C-infected individuals, with some variants in the CSF exhibiting greater resistance to broadly neutralizing antibodies than in blood variants [29]. Additionally, there were differences in the C2 region of gp120, particularly at positions 268, 281, and 283, which are essential for maintaining V3 crown integrity and facilitating coreceptor binding [29]. Similar findings were reported in a study showing that CSF-derived viruses from HIV-1 subtype B infections exhibited greater diversity, lower glycosylation, and reduced neutralization sensitivity than plasma-derived variants [58].

In contrast, previous work has demonstrated frequent genetic mixing of HIV-1 subtype C *env* sequences between plasma and CSF in this patient population, with evidence of differential predicted co-receptor usage in some patients [21, 28]. Plasma-derived variants exhibited broader tropism, including R5 and dual-tropic profiles, whereas CSF-derived variants predominantly utilized the R5 co-receptor, suggesting potential compartment-specific selective pressures. Furthermore, a study reported minor N-linked glycosylation differences at positions 301 and 160 between plasma- and CSF-derived viruses [27]. Discordant antiretroviral drug resistance profiles between compartments were also reported [26], supporting the concept of distinct replication dynamics and evolutionary pressures within the CNS.

These findings support the notion that compartmentalized viral evolution may contribute to differential neutralization sensitivity, co-receptor usage, and drug susceptibility. Despite these differences, several bnAbs in our panel, specifically VRC07-523LS, PGDM1400, PGT121, and 10E8 retained broad and potent neutralization activity against both plasma- and CSF-derived variants. This suggests that combination bnAb therapies incorporating at least two or three antibodies targeting non-overlapping epitopes may offer a promising immunotherapeutic approach, particularly for HIV-1 subtype C infections. A critical limitation of bnAb-based strategies targeting CNS reservoirs, however, is the limited ability of bnAbs to cross the blood–brain barrier (BBB). Although the bnAbs in this study demonstrated broad coverage, their therapeutic efficacy may be limited within the CNS if adequate concentrations cannot be achieved. Future research should explore strategies to enhance CNS delivery, such as using smaller antibody fragments, engineering antibodies to improve BBB penetration, or utilising nanoparticle-based delivery systems.

The limitations of our study include the small sample size and cross-sectional design, which preclude longitudinal assessment of viral evolution and escape. We analysed a limited number of clones from each compartment without limiting dilution procedures; the clones may not represent the full population of variants in plasma and CSF. All participants were ART-naïve, so the viral populations described reflect actively replicating virus rather than the latent reservoir targeted by HIV cure strategies. Latent reservoir variants in ART-suppressed individuals may differ substantially in bnAb sensitivity, and ART itself can shift the distribution of variants across compartments. Findings should therefore be extrapolated cautiously to ART-suppressed populations, and future studies should directly assess bnAb sensitivity of latent reservoir variants.

A further consideration is whether HIV-1 sequences detected in CSF accurately represent the CNS viral reservoir. CSF obtained by lumbar puncture contains predominantly cell-free viral RNA and, to a lesser extent, infected cells that have trafficked from the periphery. Whether these reflect virus actively replicating within CNS parenchymal cells, such as brain-resident macrophages and microglia, remains uncertain, as a substantial proportion of virus detected in CSF may represent peripheral blood-derived virus that has crossed the BBB rather than virus independently evolving within the CNS. However, in a post-mortem study, researchers discovered that some participants' brain tissues harboured HIV variants that were genetically distinct and remarkably resistant to certain broadly neutralising antibodies such as PG9 and PGDM1400, a finding that underscores how the blood–brain barrier may inadvertently shield resistant viruses from bnAb attack, potentially undermining therapy [30]. Unfortunately, we did not have access to brain tissues, consequently this study characterised viruses obtained from plasma and CSF samples. Further studies are required to evaluate the differences in blood and brain tissues.

Finally, throughout this study, we report conservation at potential N-linked glycosylation sites (PNGS) based on the presence of the N-X-S/T sequon in the amino acid sequence. Sequence-level conservation of a PNGS does not confirm that the PNGS is present, as glycan occupancy depends on additional cellular and structural factors, and formal confirmation requires glycoproteomic or mass spectrometry approaches, which were beyond the scope of the current study. Our conclusions regarding glycan-mediated differences in bnAb sensitivity between plasma and CSF variants should therefore be understood as inferences based on sequon conservation, pending direct experimental validation of glycan occupancy. Future studies should prioritise longitudinal sampling from larger cohorts, paired plasma-CSF analyses over time, and next-generation sequencing approaches to further elucidate the evolutionary dynamics of HIV-1 within the CNS, alongside in vivo studies investigating bnAb pharmacokinetics, CNS penetration, and antiviral efficacy.

## Conclusion

This study highlights compartment-specific differences in amino acid signatures and neutralization sensitivity between plasma- and CSF-derived HIV-1 *env* sequences. Variations were particularly evident in the CD4 binding site, V2 apex, and V3 loop, with minor differences observed in the MPER and gp120-gp41 interface. These findings emphasise the importance of considering compartmentalised viral evolution in the design of bnAb-based immunotherapies and vaccine strategies. Despite the challenges, combination bnAb regimens targeting multiple epitopes may hold significant promise in achieving effective viral suppression across anatomical compartments, including the CNS.

## Supplementary Information

**Additional file 1.**

## Data Availability

All data supporting the findings of this study are available within the paper and its Supplementary Information. The original reference describing the sequencing used in this study was included. HIV-1 Env sequences will be deposited in GenBank upon acceptance of the manuscript for publication.

## References

[1] UNAIDS. Global HIV & AIDS statistics—fact sheet. 2025.

[2] Sullivan PS, Carballo-Diéguez A, Coates T, Goodreau SM, McGowan I, Sanders EJ, et al. Successes and challenges of HIV prevention in men who have sex with men. Lancet. 2012;380(9839):388–99. 10.1016/S0140-6736(12)60955-6 PMC3670988 22819659

[3] Krishnaratne S, Hensen B, Cordes J, Enstone J, Hargreaves JR. Interventions to strengthen the HIV prevention cascade: a systematic review of reviews. Lancet HIV. 2016;3(7):e307–17. 10.1016/S2352-3018(16)30038-8 27365205

[4] Wilson D, Taaffe J, Fraser-Hurt N, Gorgens M. The economics, financing and implementation of HIV treatment as prevention: What will it take to get there? Afr J AIDS Res. 2014;13(2):109–19. 10.2989/16085906.2014.943254 25174628

[5] Shrestha R, Altice FL, Huedo-Medina TB, Karki P, Copenhaver M. Willingness to use pre-exposure prophylaxis (PrEP): an empirical test of the information-motivation-behavioral skills (IMB) model among high-risk drug users in treatment. AIDS Behav. 2017;21(5):1299–308. 10.1007/s10461-016-1650-0 PMC5380590 27990587

[6] Emmanuel G, Folayan M, Undelikwe G, Ochonye B, Jayeoba T, Yusuf A, et al. Community perspectives on barriers and challenges to HIV pre-exposure prophylaxis access by men who have sex with men and female sex workers access in Nigeria. BMC Public Health. 2020;20(1):69. 10.1186/s12889-020-8195-x PMC6964078 31941469

[7] Walker BD, Burton DR. Toward an AIDS vaccine. Science. 2008;320(5877):760–4. 10.1126/science.1152622 18467582

[8] Landais E, Moore PL. Development of broadly neutralizing antibodies in HIV-1 infected elite neutralizers. Retrovirology. 2018;15(1):61. 10.1186/s12977-018-0443-0 PMC6125991 30185183

[9] Vanhamel J, Bruggemans A, Debyser Z. Establishment of latent HIV-1 reservoirs: What do we really know? J Virus Erad. 2019;5(1):3–9. 10.1016/S2055-6640(20)30275-2 PMC6362902 30800420

[10] Astorga-Gamaza A, Buzon MJ. The active human immunodeficiency virus reservoir during antiretroviral therapy: emerging players in viral persistence. Curr Opin HIV AIDS. 2021;16(4):193–9. 10.1097/COH.0000000000000685 33973900

[11] Hessell AJ, Rakasz EG, Tehrani DM, Huber M, Weisgrau KL, Landucci G, et al. Broadly neutralizing monoclonal antibodies 2F5 and 4E10 directed against the human immunodeficiency virus type 1 gp41 membrane-proximal external region protect against mucosal challenge by simian-human immunodeficiency virus SHIVBa-L. J Virol. 2010;84(3):1302–13. 10.1128/JVI.01272-09 PMC2812338 19906907

[12] Pegu A, Yang ZY, Boyington JC, Wu L, Ko SY, Schmidt SD, et al. Neutralizing antibodies to HIV-1 envelope protect more effectively in vivo than those to the CD4 receptor. Sci Transl Med. 2014;6(243):243ra88. 10.1126/scitranslmed.3008992 PMC4562469 24990883

[13] Caskey M, Klein F, Lorenzi JC, Seaman MS, West AP Jr, Buckley N, et al. Viraemia suppressed in HIV-1-infected humans by broadly neutralizing antibody 3BNC117. Nature. 2015;522(7557):487–91. 10.1038/nature14411 PMC4890714 25855300

[14] Bar KJ, Sneller MC, Harrison LJ, Justement JS, Overton ET, Petrone ME, et al. Effect of HIV antibody VRC01 on viral rebound after treatment interruption. N Engl J Med. 2016;375(21):2037–50. 10.1056/NEJMoa1608243 PMC5292134 27959728

[15] Mendoza P, Gruell H, Nogueira L, Pai JA, Butler AL, Millard K, et al. Combination therapy with anti-HIV-1 antibodies maintains viral suppression. Nature. 2018;561(7724):479–84. 10.1038/s41586-018-0531-2 PMC6166473 30258136

[16] Corey L, Gilbert PB, Juraska M, Montefiori DC, Morris L, Karuna ST, et al. Two randomized trials of neutralizing antibodies to prevent HIV-1 acquisition. N Engl J Med. 2021;384(11):1003–14. 10.1056/NEJMoa2031738 PMC8189692 33730454

[17] Lynch RM, Boritz E, Coates EE, DeZure A, Madden P, Costner P, et al. Virologic effects of broadly neutralizing antibody VRC01 administration during chronic HIV-1 infection. Sci Transl Med. 2015;7(319):319ra206. 10.1126/scitranslmed.aad5752 PMC12366723 26702094

[18] Scheid J, Horwitz J, Bar-On Y, Kreider E, Lu C-L, CetruloLorenzi JC, et al. HIV-1 antibody 3BNC117 suppresses viral rebound in humans during treatment interruption. Nature. 2016;535:556–60. 10.1038/nature18929 PMC5034582 27338952

[19] Lu CL, Murakowski DK, Bournazos S, Schoofs T, Sarkar D, Halper-Stromberg A, et al. Enhanced clearance of HIV-1-infected cells by broadly neutralizing antibodies against HIV-1 in vivo. Science. 2016;352(6288):1001–4. 10.1126/science.aaf1279 PMC5126967 27199430

[20] Crowell TA, Colby DJ, Pinyakorn S, Sacdalan C, Pagliuzza A, Intasan J, et al. Safety and efficacy of VRC01 broadly neutralising antibodies in adults with acutely treated HIV (RV397): a phase 2, randomised, double-blind, placebo-controlled trial. Lancet HIV. 2019;6(5):e297–306. 10.1016/S2352-3018(19)30053-0 PMC6693657 31000477

[21] Sturdevant CB, Dow A, Jabara CB, Joseph SB, Schnell G, Takamune N, et al. Central nervous system compartmentalization of HIV-1 subtype C variants early and late in infection in young children. PLoS Pathog. 2012;8(12):e1003094. 10.1371/journal.ppat.1003094 PMC3531524 23300446

[22] Sturdevant CB, Joseph SB, Schnell G, Price RW, Swanstrom R, Spudich S. Compartmentalized replication of R5 T cell-tropic HIV-1 in the central nervous system early in the course of infection. PLoS Pathog. 2015;11(3):e1004720. 10.1371/journal.ppat.1004720 PMC4374811 25811757

[23] Schnell G, Joseph S, Spudich S, Price RW, Swanstrom R. HIV-1 replication in the central nervous system occurs in two distinct cell types. PLoS Pathog. 2011;7(10):e1002286. 10.1371/journal.ppat.1002286 PMC3188520 22007152

[24] Christo PP, Greco DB, Aleixo AW, Livramento JA. Factors influencing cerebrospinal fluid and plasma HIV-1 RNA detection rate in patients with and without opportunistic neurological disease during the HAART era. BMC Infect Dis. 2007;7(1):147. 10.1186/1471-2334-7-147 PMC2244630 18096083

[25] Chang C, Kangethe R, Omarjee S, Hiramen K, Gosnell B, Sojane K, et al. Relationship of HIV RNA in CSF and plasma in patients co-infected with cryptococcal meningitis. Open Forum Infect Dis. 2017;4:ofx032. 10.1093/ofid/ofx032 PMC5407210 28470016

[26] Kelentse N, Moyo S, Mogwele M, Lechiile K, Moraka NO, Maruapula D, et al. Differences in human immunodeficiency virus-1C viral load and drug resistance mutation between plasma and cerebrospinal fluid in patients with human immunodeficiency virus-associated cryptococcal meningitis in Botswana. Medicine (Baltimore). 2020;99(41):e22606. 10.1097/MD.0000000000022606 PMC7544309 33031315

[27] Kelentse N, Moyo S, Mogwele ML, Ditshwanelo D, Mokaleng B, Moraka NO, et al. HIV-1C env and gag variation in the cerebrospinal fluid and plasma of patients with HIV-associated cryptococcal meningitis in Botswana. Viruses. 2020. 10.3390/v12121404. PMC7762280 33297399

[28] Sojane K, Kangethe RT, Chang CC, Moosa MS, Lewin SR, French MA, et al. Individuals with HIV-1 subtype C infection and cryptococcal meningitis exhibit viral genetic intermixing of HIV-1 between plasma and cerebrospinal fluid and a high prevalence of CXCR4-using variants. AIDS Res Hum Retroviruses. 2018;34(7):607–20. 10.1089/AID.2017.0209 PMC6314437 29658309

[29] Stefic K, Chaillon A, Bouvin-Pley M, Moreau A, Braibant M, Bastides F, et al. Probing the compartmentalization of HIV-1 in the central nervous system through its neutralization properties. PLoS ONE. 2017;12(8):e0181680. 10.1371/journal.pone.0181680 PMC5571919 28841647

[30] Wang C, Schlub TE, Yu WH, Tan CS, Stefic K, Gianella S, et al. Landscape of human immunodeficiency virus neutralization susceptibilities across tissue reservoirs. Clin Infect Dis. 2022;75(8):1342–50. 10.1093/cid/ciac164 PMC9555844 35234862

[31] Kapoor A, Tan CS. Immunotherapeutics to treat HIV in the central nervous system. Curr HIV/AIDS Rep. 2020;17(5):499–506. 10.1007/s11904-020-00519-w PMC7492473 32671567

[32] Chang CC, Dorasamy AA, Gosnell BI, Elliott JH, Spelman T, Omarjee S, et al. Clinical and mycological predictors of cryptococcosis-associated immune reconstitution inflammatory syndrome. AIDS. 2013;27(13):2089–99. 10.1097/QAD.0b013e3283614a8d 23525034

[33] Chang CC, Kangethe R, Omarjee S, Hiramen K, Gosnell B, Sojane K, et al. Relationship of human immunodeficiency virus viral load in cerebrospinal fluid and plasma in patients co-infected with cryptococcal meningitis. Open Forum Infect Dis. 2017. 10.1093/ofid/ofx032. PMC5407210 28470016

[34] Bull M, Lee D, Stucky J, Chiu YL, Rubin A, Horton H, et al. Defining blood processing parameters for optimal detection of cryopreserved antigen-specific responses for HIV vaccine trials. J Immunol Methods. 2007;322(1–2):57–69. 10.1016/j.jim.2007.02.003 PMC1904432 17382342

[35] Williams DW, Veenstra M, Gaskill PJ, Morgello S, Calderon TM, Berman JW. Monocytes mediate HIV neuropathogenesis: mechanisms that contribute to HIV associated neurocognitive disorders. Curr HIV Res. 2014;12(2):85–96. 10.2174/1570162x12666140526114526 PMC4351961 24862333

[36] Rousseau CM, Birditt BA, McKay AR, Stoddard JN, Lee TC, McLaughlin S, et al. Large-scale amplification, cloning and sequencing of near full-length HIV-1 subtype C genomes. J Virol Methods. 2006;136(1–2):118–25. 10.1016/j.jviromet.2006.04.009 16701907

[37] Wei X, Decker JM, Wang S, Hui H, Kappes JC, Wu X, et al. Antibody neutralization and escape by HIV-1. Nature. 2003;422(6929):307–12. 10.1038/nature01470 12646921

[38] Montefiori DC. Evaluating neutralizing antibodies against HIV, SIV, and SHIV in luciferase reporter gene assays. Curr Protoc Immunol. 2005;Chapter 12:12.1.1–1.7. 10.1002/0471142735.im1211s64 18432938

[39] Ndlovu B, Gounder K, Muema D, Raju N, Hermanus T, Mthethwa Q, et al. Envelope characteristics in individuals who developed neutralizing antibodies targeting different epitopes in HIV-1 subtype C infection. Virology. 2020;546:1–12. 10.1016/j.virol.2020.03.003 PMC8186003 32275203

[40] Montefiori DC. Measuring HIV neutralization in a luciferase reporter gene assay. In: Prasad VR, Kalpana GV, editors. HIV protocols. Totowa: Humana Press; 2009. p. 395–405. 10.1007/978-1-59745-170-3_26 19020839

[41] Gray ES, Madiga MC, Hermanus T, Moore PL, Wibmer CK, Tumba NL, et al. The neutralization breadth of HIV-1 develops incrementally over four years and is associated with CD4+ T cell decline and high viral load during acute infection. J Virol. 2011;85(10):4828–40. 10.1128/JVI.00198-11 PMC3126191 21389135

[42] Kwong PD, Wyatt R, Robinson J, Sweet RW, Sodroski J, Hendrickson WA. Structure of an HIV gp120 envelope glycoprotein in complex with the CD4 receptor and a neutralizing human antibody. Nature. 1998;393(6686):648–59. 10.1038/31405 PMC5629912 9641677

[43] Pancera M, Zhou T, Druz A, Georgiev IS, Soto C, Gorman J, et al. Structure and immune recognition of trimeric pre-fusion HIV-1 Env. Nature. 2014;514(7523):455–61. 10.1038/nature13808 PMC4348022 25296255

[44] Brunel FM, Zwick MB, Cardoso RM, Nelson JD, Wilson IA, Burton DR, et al. Structure-function analysis of the epitope for 4E10, a broadly neutralizing human immunodeficiency virus type 1 antibody. J Virol. 2006;80(4):1680–7. 10.1128/JVI.80.4.1680-1687.2006 PMC1367132 16439525

[45] Ofek G, Tang M, Sambor A, Katinger H, Mascola JR, Wyatt R, et al. Structure and mechanistic analysis of the anti-human immunodeficiency virus type 1 antibody 2F5 in complex with its gp41 epitope. J Virol. 2004;78(19):10724–37. 10.1128/JVI.78.19.10724-10737.2004 PMC516390 15367639

[46] Gray ES, Meyers T, Gray G, Montefiori DC, Morris L. Insensitivity of paediatric HIV-1 subtype C viruses to broadly neutralising monoclonal antibodies raised against subtype B. PLoS Med. 2006;3(7):e255. 10.1371/journal.pmed.0030255 PMC1502151 16834457

[47] Archary D, Gordon ML, Green TN, Coovadia HM, Goulder PJ, Ndung’u T. HIV-1 subtype C envelope characteristics associated with divergent rates of chronic disease progression. Retrovirology. 2010;7:92. 10.1186/1742-4690-7-92 PMC2992043 21050445

[48] Huang J, Ofek G, Laub L, Louder MK, Doria-Rose NA, Longo NS, et al. Broad and potent neutralization of HIV-1 by a gp41-specific human antibody. Nature. 2012;491(7424):406–12. 10.1038/nature11544 PMC4854285 23151583

[49] Schiffner T, Phung I, Ray R, Irimia A, Tian M, Swanson O, et al. Vaccination induces broadly neutralizing antibody precursors to HIV gp41. Nat Immunol. 2024;25(6):1073–82. 10.1038/s41590-024-01833-w PMC11147780 38816615

[50] Kim AS, Leaman DP, Zwick MB. Antibody to gp41 MPER alters functional properties of HIV-1 Env without complete neutralization. PLoS Pathog. 2014;10(7):e1004271. 10.1371/journal.ppat.1004271 PMC4110039 25058619

[51] Falkowska E, Le KM, Ramos A, Doores KJ, Lee JH, Blattner C, et al. Broadly neutralizing HIV antibodies define a glycan-dependent epitope on the prefusion conformation of gp41 on cleaved envelope trimers. Immunity. 2014;40(5):657–68. 10.1016/j.immuni.2014.04.009 PMC4070425 24768347

[52] Rademeyer C, Korber B, Seaman MS, Giorgi EE, Thebus R, Robles A, et al. Features of recently transmitted HIV-1 clade C viruses that impact antibody recognition: implications for active and passive immunization. PLoS Pathog. 2016;12(7):e1005742. 10.1371/journal.ppat.1005742 PMC4951126 27434311

[53] Wagh K, Seaman MS, Zingg M, Fitzsimons T, Barouch DH, Burton DR, et al. Potential of conventional & bispecific broadly neutralizing antibodies for prevention of HIV-1 subtype A, C & D infections. PLoS Pathog. 2018;14(3):e1006860. 10.1371/journal.ppat.1006860 PMC5854441 29505593

[54] Mkhize NN, Yssel AEJ, Kaldine H, van Dorsten RT, Woodward Davis AS, Beaume N, et al. Neutralization profiles of HIV-1 viruses from the VRC01 antibody mediated prevention (AMP) trials. PLoS Pathog. 2023;19(6):e1011469. 10.1371/journal.ppat.1011469 PMC10337935 37384759

[55] Ndlovu B, Gounder K, Zikhali N, Singh L, Ntshangase N, Gumede N, et al. Distinct neutralization sensitivity between adult and infant transmitted/founder HIV-1 subtype C viruses to broadly neutralizing monoclonal antibodies. PLoS Pathog. 2025;21(6):e1013245. 10.1371/journal.ppat.1013245 PMC12225807 40549817

[56] Zhou T, Zhu J, Wu X, Moquin S, Zhang B, Acharya P, et al. Multidonor analysis reveals structural elements, genetic determinants, and maturation pathway for HIV-1 neutralization by VRC01-class antibodies. Immunity. 2013;39(2):245–58. 10.1016/j.immuni.2013.04.012 PMC3985390 23911655

[57] Wang Q, Liu L, Ren W, Gettie A, Wang H, Liang Q, et al. A single substitution in gp41 modulates the neutralization profile of SHIV during in vivo adaptation. Cell Rep. 2019;27(9):2593-607.e5. 10.1016/j.celrep.2019.04.108 31141685

[58] Pillai SK, Pond SLK, Liu Y, Good BM, Strain MC, Ellis RJ, et al. Genetic attributes of cerebrospinal fluid-derived HIV-1 env. Brain. 2006;129(7):1872–83. 10.1093/brain/awl136 16735456

[59] Sok D, Pauthner M, Briney B, Lee JH, Saye-Francisco KL, Hsueh J, Ramos A, Le KM, Jones M, Jardine JG, Bastidas R, Sarkar A, Liang CH, Shivatare SS, Wu CY, Schief WR, Wong CH, Wilson IA, Ward AB, Zhu J, Poignard P, Burton DR. A Prominent Site of Antibody Vulnerability on HIV Envelope Incorporates a Motif Associated with CCR5 Binding and Its Camouflaging Glycans. Immunity. 2016 Jul 19;45(1):31-45. 10.1016/j.immuni.2016.06.026. PMID: 27438765; PMCID: PMC4990068. PMC4990068 27438765

